# Analysis of Immunological Memory for John Cunningham Virus in a Mexican Population of Patients with Multiple Sclerosis Under Treatment

**DOI:** 10.3390/biomedicines12122737

**Published:** 2024-11-29

**Authors:** Silvia García, Adriana García-Martell, Sandra Quiñones-Aguilar, Sergio Sauri-Suárez, Mario Antonio Téllez González, Guillermo García-Castillo, Juan Antonio Suárez-Cuenca, Christian Gabriel Toledo-Lozano, Paul Mondragón Terán, Sofia Lizeth Alcaraz-Estrada

**Affiliations:** 1 Clinical Research Department, National Medical Center “20 de Noviembre” Instituto de Seguridad y Servicios Sociales de los Trabajadores del Estado, Mexico City 03100, Mexico; rolasil@yahoo.com.mx (S.G.); adrimartell@hotmail.com (A.G.-M.); suarej05@gmail.com (J.A.S.-C.); drchristiantoledo@gmail.com (C.G.T.-L.); 2 Neurology Department, National Medical Center “20 de Noviembre” Instituto de Seguridad y Servicios Sociales de los Trabajadores del Estado, Mexico City 03100, Mexico; quinonessa@gmail.com (S.Q.-A.); sssauri@aol.com (S.S.-S.); 3 Biomedical Research Division, National Medical Center “20 de Noviembre” Instituto de Seguridad y Servicios Sociales de los Trabajadores del Estado, Mexico City 03100, Mexico; mario.atg91@gmail.com; 4 Special Testing Laboratory, National Medical Center “20 de Noviembre” Instituto de Seguridad y Servicios Sociales de los Trabajadores del Estado, Mexico City 03100, Mexico; guillermo.garcia@uw.edu.mx; 5 Centro de Investigación en Ciencia Aplicada y Tecnología Avanzada Unidad Morelos, Instituto Polítecnico Nacional, Mexico City 03100, Mexico; p.mondragonteran@gmail.com; 6 Virological Analysis and Reference Unit, National Medical Center “20 de Noviembre” Instituto de Seguridad y Servicios Sociales de los Trabajadores del Estado, Mexico City 03100, Mexico

**Keywords:** multiple sclerosis, JCV, antibody index, HET

## Abstract

**Background/Objectives**: Multiple sclerosis (MS) is a disease characterized by demyelination and axonal damage of the central nervous system. Despite the observed benefits, highly effective treatment (HET)-based therapy has adverse effects, which include an increased risk of developing progressive multifocal leukoencephalopathy (PML). Additionally, the risk grows if the patient has antibodies for the John Cunningham virus (JCV). The appearance of PML is rare, and only one report has been found in Mexico. The objective of this research was to determine and analyze the immunological memory for JCV in a population of Mexican patients with MS under treatment. **Methods**: All participants underwent a complete medical history and neurological evaluation. Once they signed their informed consent, a blood sample was taken to determine if antibodies against JCV were present in their serum. **Results:** In total, 121 MS patients were analyzed, and the population consisted of 62.8% women and 37.2% men with an average age of 42.28. The three most common HETs received by the participants were natalizumab (67.76%), followed by teriflunomide and fingolimod. **Conclusions**: The seropositivity was 62.8%, and in this group, the average duration of disease evolution was 152.33 ± 93.37 months. Natalizumab was the most used HET, and despite this, only a positive association between a positive JCV antibody index with duration of fingolimod and history of depression was found. Also, a positive correlation of the JCV Ab index within the forms of SPMS and PPMS compared to RRMS was observed. No differences were observed between populations, type, and duration of MS.

## 1. Introduction

Multiple sclerosis (MS) is a chronic, progressive, immune-mediated disease characterized by demyelination and axonal damage of the central nervous system (CNS). It mainly affects women between 20 and 40 years of age [1]; its incidence varies from 2 to 9.6 per 100,000 inhabitants per year; the prevalence in the USA in 2010 was 309.2/100,000 inhabitants; and the female–male ratio was 2.8:1. In 2017, the prevalence increased by 9% compared to 2010 (338/100,000 inhabitants) [2].

MS is considered to have a complex, polygenic, and multifactorial etiology. More than 200 susceptibility variants have been proposed at the MHC-encoding loci 6p21.3 [3]. The HLA-DRB1*1501 allele is the variant most associated with the risk of developing MS in different populations, with heritability being ~30% in Northern Europe, 15% in Italy, and 6% in France. This trend represents the impact of the environment on a genetically predisposed host [4]. This predisposition is confirmed by the discrepancy in the risk of MS in the general population of 0.3% and its tenfold increase in first-degree relatives [1].

Inflammation in MS is a prominent phenomenon and is attributed to the adaptive autoimmune response mediated by T and B lymphocytes against the CNS [5]. Neuroaxonal degeneration is probably the main biological target of progression; however, chronic inflammation contributes to tissue loss and disability [6]. In the last 30 years, so-called disease-modifying therapies (DMTs) have been developed, with the main purpose to reduce early disease activity and thus reduce long-term disability [7].

Among the DMTs, monoclonal antibodies (mAbs) are known as highly effective treatments (HETs) [8]; however, they have the highest risks [9]. Natalizumaf, a recombinant humanized IgG4κ antibody selective for α4-integrin (VLA-4) that prevents leukocyte migration through the blood–brain barrier (BBB) [10], reduces relapses by 67%, reduces new MRI lesions by 83%, and limits confirmed disability progression (DRP) by 42%. It is used as a monotherapy for MS; however, among the serious adverse effects is the increased risk of developing progressive multifocal leukoencephalopathy (PML), where the estimates vary widely from 1 in 90 [11] to 0.7/1000 treated patients. What remains consistent is that the risk increases up to 2.7 times if the host has antibodies for the John Cunningham virus (JCV) [12]. Other mAbs have been reported to be associated with the development of PML, such as rituximab, daclizumab, alemtuzumab, efalizumab, and ofatumumab [13], and non-mAb DMTs such as dimethyl fumarate [14] and fingolimod [15,16]. The common denominator of these HETs is a decrease in immune response.

PML is a rare, demyelinating, inflammatory, subacute CNS disease related to JCV infection, with a mortality rate of 30% [17]. JCV is a polyomavirus that causes asymptomatic infection in the early stages of life in immunocompetent populations and remains latent throughout the life of the host; when JCV is reactivated, it is because of immunological deterioration and produces a neurological condition characterized by cognitive and behavioral changes, language disorders, weakness, and visual deficits. In Mexico, our group published the only report on progressive multifocal leukoencephalopathy (PML) in a patient with multiple sclerosis (MS) treated with natalizumab [18].

Based on what has been described, the hypothesis of this study is that the prevalence of immunological memory for the JC virus (JCV) in Mexican MS patients treated with natalizumab is either equal to or higher than that in underdeveloped countries (60%) and is positively correlated with factors such as disease duration and type of treatment. The aim of this research was to assess, determine, and analyze the immunological memory for JCV in a population of Mexican MS patients undergoing treatment.

## 2. Materials and Methods

This was an analytical study carried out on patients treated at a reference hospital center for MS patients in Mexico City. The protocol was submitted and approved by the center’s research, biosafety research, and ethics committees. All participants signed a written informed consent letter authorizing their participation and received a privacy notice. The cases were Mexican patients diagnosed with MS according to the 2017 McDonald criteria [19], which consider clinical relapses, MRI findings demonstrating dissemination in space (lesions in at least two regions: periventricular, juxtacortical, infratentorial, or medullary) and time (new or simultaneous enhancing and non-enhancing lesions), as well as the presence of oligoclonal bands in the cerebrospinal fluid (CSF).

The patients were under treatment, of both sexes, and aged 12 years or older. All participants underwent a complete medical history, Expanded Disability Status Scale (EDSS) classification, and neurological evaluation. Once they met the selection criteria, they were given a structured questionnaire, and a 10 mL venous blood sample was taken from the forearm of their non-dominant side to determine the level of anti-JCV antibodies using a two-step ELISA or double-ELISA (screening and confirmatory) assay. This determination was carried out at the Virological Analysis and Reference Unit (UARVI). The results were stratified according to the UARVI specifications in accordance with the Kit: positive (anti-JCV antibody index > 0.4); negative (anti-JCV antibody index < 0.2); and indeterminate (anti-JCV antibody index between >0.2 and <0.4).

The socio-demographic and clinical data were obtained from the electronic medical record available to the researchers who participated in the study.

The participants were divided into three groups according to the result of the anti-JCV antibody index (negative, indeterminate, and positive). Descriptive statistics were performed to understand the behavior of the cohort and inferential statistics appropriate to the type of variable. Those that did not describe a normal curve were standardized using the statistical package according to their distribution and the presence or absence of values equal to or less than 0. *p* < 0.05 was accepted as a significant difference. The analysis was performed with the SPSS-25 program.

## 3. Results

Samples from 121 MS patients were analyzed: 76 women (62.8%) and 45 (37.2%) men. The mean age was 42.28 ± 10.62. The socio-demographic characteristics are described in Table 1.

**Figure 1:**
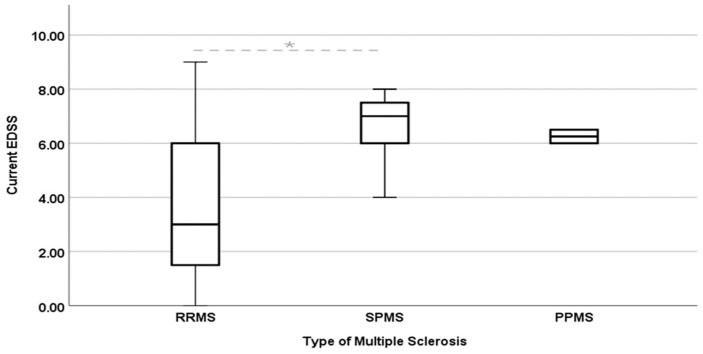
EDSS of patients with MS. The Chi-squared test revealed a significant moderate association between EDSS and the type of multiple sclerosis, χ^2^ = 59.18, N 121, *p* = 0.005, 95% CI: 3.14–4.08 RRMS, 6.3–7.2 SPMS, 3.07–9.4 PPMS, and V of Crammer = 0.495. Meanwhile, the Kruskal–Wallis test indicated a significant difference on the current EDSS across the type of multiple sclerosis, H(2) = 32.15, *p* = 0.005. Post hoc pairwise comparisons using the Dunn test revealed differences between RRMS and SPMS, *p* = *0.0001, but no significant differences were found between RRMS and PPMS, *p* = 0.170, and PPMS and SPMS, *p* = 0.626 (EDSS from 0 to 9).

The analysis of the clinical characteristics of the patients determined the distribution by clinical type of the disease as follows: 81% presented with RRMS (Relapsing–Remitting MS) (67.34% women and 32.65% men, with an average age of 30.68 ± 9.65 years, median 30 years (max. 50–min. 12)); 17.35% with SPMS (Secondary Progressive MS) (47.61% women and 52.38% men, with a mean age of 33.62 ± 11.27, median 32 years (max. 59–min. 17), average duration of evolution of MS of 176 ± 87 months, median 168 (max. 360–min. 24)); and two patients with PPMS (Primarily Progressive MS) (both men aged 19 and 30 and with a duration of 96 and 180 months) (Table 2). Additionally, we observed a marked difference in the EDSS of the different clinical types of MS that the patients presented (Figure 1). EDSS is a disability scale for MS. RRMS has a lower range of EDSS (median close to 2). This reflects relatively less disability. On the other hand, SPMS has a higher EDSS (median near 6), which is clinically consistent, as SPMS represents a more advanced phase with greater accumulated disability. Significant differences in disability between RRMS and SPMS (*p* = 0.0001) were confirmed by statistical analyses such as the Chi-squared and Kruskal–Wallis tests, consistent with the natural history of the disease. These analyses establish a statistical relationship between clinical severity and type of MS, indicating that RRMS is less disabling than SPMS in function.

The HETs received during the study were natalizumab in 67.76% of patients, teriflunomide in 11.57%, fingolimod in 9.09%, glatiramer acetate in 2.47%; cladribine and ocrelizumab in 1.65% each; and azathioprine, immunoglobulin G, and interferons in 0.826% each (Table 3, Table 4 and Table 5).

The JCV index was determined in all participants, and they were classified into three groups, as described in the Materials and Methods section. The first group (negative) consisted of 31 patients, 21 (67.74%) women and 10 (32.25%) men: 26 (83.9%) patients under treatment with natalizumab; 2 (5.45%) with teriflunomide; and 1 each with ocrelizumab, azathioprine, and interferon (Table 3).

The second group (intermediate), composed of patients with an index between 0.2 and less than 0.4, consisted of 14 patients, 10 (71.42%) women and 4 (28.57%) men: 12 (85.7%) patients were treated with natalizumab, 1 with fingolimod, and another with glatiramer acetate (Table 4).

The third group, classified as positive, consisted of 76 patients with MS and whose antibody index against JCV was 0.4 or higher; 46 (60.52%) were women and 30 (39.47%) men. The treatment they were receiving at the time of the study was as follows: 2 (2.62%) patients were receiving treatment with glatiramer acetate, 4 (5.26%) received alemtuzumab, 2 (2.62%) received cladribine, 10 (13.15%) received fingolimod, 44 (57.89%) patients received natalizumab, 12 patients received teriflunomide, and 1 patient each received ocrelizumab and immunoglobulin G (Table 5). It is important to note that the treatments with azathioprine and immunoglobulin G used in this group of patients were administered off-label for MS. These decisions reflect the clinical judgment of the treating physician, informed by available evidence in specific cases. According to the type of MS, within these group, 75% of patients presented with RRMS, 22.36% of patients with SPMS, and 2.63% of the patients with PPMS. In this group, the therapies administered include natalizumab, alemtuzumab, and fingolimod, all of which are classified as HETs according to the literature [20]. In contrast, treatments such as glatiramer acetate, interferons, and teriflunomide are not classified as HETs but may be used in specific cases based on the individual clinical needs of the patient.

**Figure 2:**
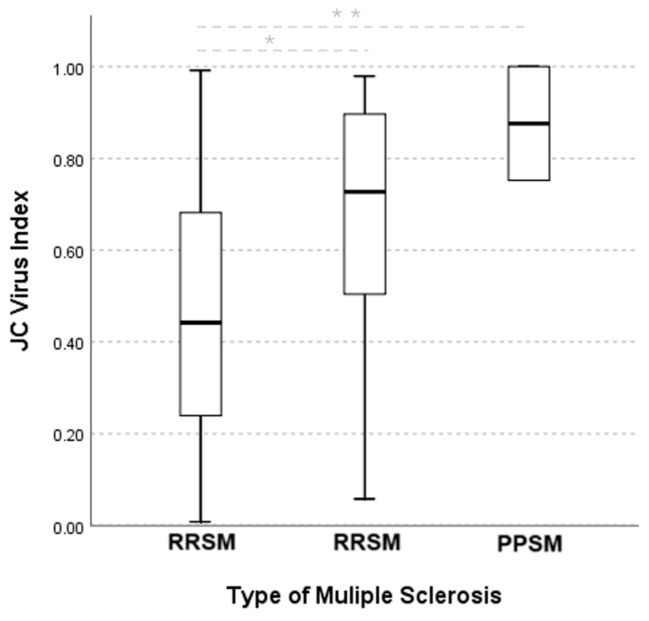
JCV antibody index and clinical form of MS. The Chi-squared test revealed a significant high association between JC virus index and the type of multiple sclerosis, χ^2^ = 219.14, N 121, *p* = 0.007, 95% CI: 0.4–0.51 RRMS, 95% CI: 0.52–0.79 SPMS, 95% CI: −0.75–1 PPSM, and V of Crammer = 0.95. Meanwhile, the Kruskal–Wallis test indicated a significant difference on the JC virus index across the type of multiple sclerosis, H(2) = 11.32, *p* = 0.003. Post hoc pairwise comparisons using the Dunn test revealed differences between RRMS and SPMS, *p* = 0.005 *, and RRMS and PPMS, *p* = 0.046 **, but no significant differences between SPMS and PPMS, *p* = 0.314.

In the association tests, we found significant relationships between IgG positivity and certain clinical conditions: cognitive impairment, history of depression, and absence of other neurological diseases. A correlation analysis showed that the JCV antibody index was correlated with a history of depression and the duration of fingolimod use (Table 6). These associations suggest that the existence of antibodies against JCV could impact inflammation or neurodegenerative mechanisms in the brain, thereby exacerbating cognitive and emotional disorders. Virus-induced neuroinflammation, in addition to the immunologic effects of the treatments (i.e., natalizumab), may contribute to these clinical conditions. Additionally, the JCV antibody index was significantly associated with the clinical form of MS, particularly between RRMS and SPMS and between RRMS and PPMS (Figure 2). This may reflect an increased susceptibility to viral reactivation and immune changes in patients with more progressive disease, with implications for therapy selection and clinical monitoring. These analyses highlight the relevance of JCV seropositivity not only for PML risk but also for the clinical parameters of MS course.

## 4. Discussion

MS remains a disease that faces many challenges, and despite the significant progress in treatment with encouraging response rates of around 70% with DMTs [21], some limitations and complications of these treatments have been identified.

Additionally, viruses in MS have been identified at different stages of the disease from development to evolution and treatment of the disease, for example, the Epstein–Barr virus (EBV), whose infection increases the risk of developing MS by 26.5% (95% CI: 3.7–191.6) [22]. It is speculated that, after acute infection, EBV remains quiescent in peripheral blood B lymphocytes and lymph nodes [23], which in turn present antigens like myelin and, by interacting with T lymphocytes, enters the CNS with subsequent immune attacks and inflammation [24]. In addition, EBV infection bound to transcription factors confers a higher risk of developing autoimmune diseases [25].

JCV appeared on the MS scene from the use of DMTs, particularly mAc. JVC causes an asymptomatic primary infection in the early stages of life. Its access is through the digestive or respiratory tract until it reaches epithelial cells of the kidney, bone marrow, spleen, and lymphocytes cells, where it remains throughout the life of the host [26]. Although first contact with JCV usually occurs in childhood, it can also happen in adulthood, so seropositivity increases with age. It has been estimated that each year, 3% of the seronegative population becomes positive [27].

Under immunosuppressive conditions, JCV causes a disease known as PML, described by Åström in 1958, although the virus was not identified until 1971 [28]. PML is a multifocal and progressive white matter disease of the CNS where the host suffers from severe suppression of cellular immunity during its development. Primary infection is caused by the “archetype” virus, whose non-coding region or hypervariable regulatory region [29] does not replicate effectively in the brain; however, in immunosuppressed individuals, active viral replication is observed with rearrangements in the viral genome that generate neurotropic variants that actively replicate in glial cells. When JCV reaches the CNS, it lytically infects glial cells, particularly oligodendrocytes [30].

With the advent of DMTs, particularly the use of mAb, the presence of immunological memory for JCV and viruses are of interest in MS patients due to the risk of developing PML; in our cohort, we found that the positivity of immunological memory for JCV was 62.8%. Paz et al. [31], in their systematic review of 1664 articles, which included Eurasia, Canada, and the USA, observed in a total of 29,319 patients with MS and Neuromyelitis Optica an average of 57.1% seropositivity, slightly lower than that observed in the present study. In this systematic review, two Portuguese, two Austrian, one German, one Belgian, one Korean, one Dutch, and one Turkish study showed prevalences of 60% or more, and it is worth noting that the sociodemographic heterogeneity of these countries is striking. However, seropositivity for anti-JCV antibodies and its association with the use of natalizumab were not consistent across the studies reviewed. This variability may indicate a potential bias, as it is not typically observed in larger samples, and in our case, it was not identified.

In another meta-analysis of 17 independent cohorts, where 1221 patients with MS were treated with natalizumab, it was observed that on average, 10.80% of JCV-seronegative patients became positive one year after treatment. When this conversion is at low index values, there is a high probability of reversion over time, but patients with high index values almost never experience reversion, so the conversion rate by natalizumab is estimated to be three to four times higher than the biological conversion by age [32].

In a German cohort of 803 MS patients [33], the prevalence of anti-JCV antibody positivity was 67.9%. The male–female ratio was 81.7% and 64%, respectively (*p* < 0.001, OR = 2.51; CI: 1.65–3), which we did not determine in our study. Those who lived in cold regions had a significantly higher prevalence of positivity (*p* = 0.043 and OR = 1.86; IC 1.02–3.39) [32]. Additionally, they found no differences with the type of MS, dose and duration of immunosuppressive drugs used, and positivity of anti-JCV antibodies or the index. Unlike Germany, Mexico has few cold areas, which are located in the north of the country; moreover, the patients studied were mostly residents of the center of the country, which is characterized by warm temperatures, so this variable was not considered. In the German cohort, they observed that, among those whose MS onset was between 28 and 37 years of age, there was more positivity compared with in those whose onset was between 18 and 27 years of age (*p* = 0.02; OR = 1.85; CI: 1.09–3.14). In contrast, in another study, age at disease onset was not associated with JCV positivity, but the age of the patients was, suggesting that exposure to a wild-type virus is the underlying phenomenon for seroconversion in this study group and perhaps in central Mexico [34].

A Brazilian study [35] where 1501 blood samples from 1102 MS patients with various treatment regimens were analyzed for five years, 76% were women, with a median age of 38 years. The initial seropositivity was 55.3%, the disease was more common among men (*p* = 0.001), and age did not influence the result (r = 0.115). Seroconversion occurred in 18.5%, over a mean period of 22 months (0.84% per month and 10.09% per year), and an overall seropositivity of 57.1% was achieved, with no difference from the baseline seropositivity (*p* = 0.09). This increase was significantly higher than that estimated by Schwab et al., ~0.48%/year [32], perhaps because the Brazilian population has more exposure to JCV. The Brazilian population might share more socioeconomic similarities with our cohort, however, and the distance in positivity between 55.3% and 62.8% could be explained by population or geographic differences, highlighting that the patients in the present study are significantly younger (30 vs. 38 years), so we would expect a higher prevalence in Brazilians. Auer et al. [26] estimated an increase of 3% per year; based on this estimate, for our cohort, if there is no change in the rate of increase, we would expect 4.7 patients to acquire MS per year, which equates to 38 patients in 8 years, and a seropositivity of 94% would be reached, which opens up an area of research for this population difference. In Brazil, Fragoso et al. [36] studied 168 patients with MS based on type of MS and treatment with natalizumab for JVC using PCR and found positivity in 51.2%, a very concerning figure that deserves to be studied.

Bozice et al. [37] observed in 7724 patients with MS from 10 countries in Europe, Canada, and Austria (median age of 43 years, 71% women, 94% white population, with various treatments and all forms of the disease) an overall prevalence of anti-JCV antibodies of 57.1%, which was associated with increasing age, being male, and living in Australia, Norway, and Portugal (*p* < 0.0001). There was no difference with disease duration, type of MS, number of relapses, treatments, and treatment duration.

Olson et al. [38] found in 378 patients with RRMS a mean prevalence of anti-JCV antibodies of 57.6%, and it was associated with older age (*p* = 0.030) and with treatment with natalizumab (*p* < 0.001); in our cohort, prevalence of anti-JCV antibodies was also associated with older age of the patients, but we did not find any association with the various treatments.

Bhan et al. [39] recruited 4198 MS patients from Quebec, Ontario, the Atlantic provinces (Nova Scotia, New Brunswick, and Newfoundland), the western provinces (Manitoba, Saskatchewan, Alberta, and British Columbia), and the rest of Canada. Seropositivity was determined by ELISA (two steps). Overall seropositivity was 56.3%, associated with age (*p* = 0.0001), being male (*p* = 0.005), and residing in Quebec (*p* = 0.005). No differences were observed between populations, type, and duration of MS.

Sá et al. [40] analyzed 655 MS patients in Portugal, and the overall seroprevalence of anti-JCV antibodies was 60.8% and was associated with older age (*p* = 0.030); it was surprisingly lower in patients treated with natalizumab 48.4% vs. 68.3% (*p* < 0.001), a finding that the researchers attributed to a selection bias, since patients with a positive result do not usually receive natalizumab.

A multicenter study in five regions of Poland included 1405 MS patients whose positivity for JVC Ab was 65.8% [41]; these authors found that there was a correlation of the JCV Ab index within the forms of SPMS and PPMS compared to RRMS patients, a finding also observed in our study and whose explanation is not clear. As expected, more than half of patients with PML are seropositive for JCV before the onset of the disease, but it is relevant that higher levels of antibodies have been detected before the diagnosis of PML compared to those who do not develop it, and in patients treated with natalizumab, an increase in antibodies has been observed shortly before or coinciding with the development of PML attributed to JCV reactivation [29]. However, the function and interpretation of anti-JCV antibodies in the evolution of the disease remain to be elucidated.

Regarding the significant association between a positive index and a history of depression, various investigations have confirmed the important role that glia plays in the development of depression. There is evidence that suggests that the development of depression may be strongly related to viral infections (herpes simplex-1, varicella zoster, and Epstein–Barr virus, among others [42]) that usually generates damage to glial cells (astrocytes, oligodendrocytes, and microglia), which causes alterations in neurotransmission; decreases the synthesis of monoamines; and increases the level of cytokines, the consequent neuroinflammation and mitochondrial damage, excitotoxicity, and oxidative stress that eventually results in depression [43]. Along with direct cell infection, other mechanisms of viral infection-induced depression have been proposed, including immune imbalance. This is carried out through chronic or excessive inflammation, driven by the stimulation of a robust production of cytokines and chemokines. This response can become dysregulated, leading to an altered hypothalamic–pituitary–adrenal axis, activation of microglia and astrocytes, and modification of metabolic pathways that changes the neurotransmitter system and synaptic function. All these mechanisms ultimately contribute to and play a crucial role in mood regulation [44].

Based on the close relationship between the different types of viruses and depression, we could consider positivity for JCV in the list of associated viruses. It was very relevant that the correlation between the JCV Ab index and depression was negative, which suggests that it is more related to the primary infection than to the magnitude of the host response.

Finally, it is possible that the behavior of JCV in people with MS differs and that this difference increases with the use of treatment, particularly highly effective disease-modifying treatments, whose common denominator is a reduction in the immune response.

Our study was limited by the sample size, so in the future, it would be interesting not only to increase the sample size but also to provide long-term follow-up to determine seroconversion and its relationship with the treatments that patients receive and additionally add studies that determine JCV infection at the time of treatment. Other limitations to consider are that control treatments were not performed, since this is a third-level hospital and this type of treatment is only given to people with MS. Moreover, the study population was taken from the center of the country, which limits the representation of regions with other climates or where the prevalence of the JCV may vary; therefore, not including these populations may have impacted the possible generalization of the findings. This was a cross-sectional study, which limits the evaluation of changes in antibody levels and disease progression in the people who participated. Other viruses that may contribute to the pathogenesis of MS such as Epstein–Barr virus were also not considered.

## 5. Conclusions

The seropositivity was 62.8%, and in this group, the average duration of disease evolution was 152.33 ± 93.37 months. Natalizumab was the most used HET, and despite this, only a positive association between a positive JCV antibody index with duration of fingolimod and history of depression was found. Also, a positive correlation of the JCV Ab index within the forms of SPMS and PPMS compared to RRMS was observed. No differences were observed between populations, type, and duration of MS.

## Figures and Tables

**Table 1:** Demographic characteristics of the participants.

	Patients with MS
Number of Participants	121
Women	76 (62.8%)
Age	42.28 ± 10.62
Educational Level	
Postgraduate	12
Bachelor’s Degree	69
High School or Technical Degree	34
Secondary School	3
Primary School	3
Marital Status	
Single	61
Married	44
Free Union	8
Divorced	7
Widowed	1
Place of Residence	
Mexico City and Metropolitan Area	86
Rest of the Country	35
Occupation	
Professionals	17
Public Sector Administrators	33
Home	16
Pensioners	15
Teachers	10
Unemployed	14
Students	4
Police	3
Merchants	2
Other	7

**Table 2:** Clinical characteristics of patients with MS.

	*RRMS	**SPMS	***PPMS
Number of patients	98 (81%)	21 (17.35%)	2 (1.65%)
Duration of the disease in months			
Mean	139 ± 88	181 ± 88	96
Median	120 (408–24)	180 (306–24)	180
Women	66 (67.34%)	10 (47.61%)	0
Age in years			
Mean	30.68 ± 9.65	33.64 ± 11.27	19
Median	30 (50–12)	32 (59–17)	30
Cognitive impairment	42 (41%)	14 (66%)	2 (100%)
History of depression	29 (29.5%)	13 (62%)	1 (50%)
Negative history of another neurological disease	90 (92%)	20 (95.2%)	2 (199%)
EDSS			
0	6	-	-
1.0	13	-	-
1.5	15	-	-
2.0	3	-	-
2.5	6	-	-
3.0	8	-	-
3.5	2	-	-
4.0	4	1	-
4.5	2	-	-
5.0	5	-	-
5.5	4	1	-
6.0	10	4	1
6.5	15	3	1
7.0	3	5	-
7.5	1	3	-
8.0	-	4	-
8.5	-	-	-
9.0	1	-	-

*RRMS, Relapsing–Remitting Multiple Sclerosis; **SPMS, Secondary Progressive Multiple Sclerosis; ***PPMS, Primary Progressive Multiple Sclerosis; EDSS, Expanded Disability Status Scale; Chi squared test, *p* = 0.005; Rho 0.51, *p* = 0.0001. Frequencies; mean; median; Chi-squared test, *p* = 0.005; and Rho Spearman 0.51, *p* = 0.0001 (disability grade (EDSS)/SM type) were used.

**Table 3:** Patients with negative anti-JCV antibody classification.

Sex	Age in Years	Type of MS	Evolution in Months	Current Treatment	EDSS	JCV Index	
Woman	28	RRMS	108	Natalizumab	2.5	0.07	Negative
Woman	66	RRMS	228	Natalizumab	6.5	0.08	Negative
Woman	33	RRMS	216	Natalizumab	6.0	0.08	Negative
Woman	50	RRMS	96	Natalizumab	2.5	0.09	Negative
Man	60	SPMS	180	Interferon	8.0	0.1	Negative
Woman	51	RRMS	336	Natalizumab	5.0	0.1	Negative
Man	25	RRMS	168	Natalizumab	1.5	0.1	Negative
Woman	56	RRMS	312	Azathioprine	7.0	0.1	Negative
Woman	59	RRMS	180	Teriflunomide	2.5	0.1	Negative
Woman	42	RRMS	144	Ocrelizumab	6.0	0.12	Negative
Woman	27	RRMS	24	Natalizumab	2.5	0.12	Negative
Woman	46	RRMS	300	Natalizumab	3.0	0.12	Negative
Man	28	RRMS	120	Natalizumab	3.0	0.12	Negative
Woman	50	RRMS	132	Natalizumab	6.5	0.12	Negative
Man	37	RRMS	48	Natalizumab	6.0	0.13	Negative
Woman	48	RRMS	72	Natalizumab	2.5	0.13	Negative
Woman	38	SPMS	84	Natalizumab	7.0	0.14	Negative
Woman	33	RRMS	24	Natalizumab	1.5	0.14	Negative
Man	26	RRMS	60	Natalizumab	1.0	0.14	Negative
Woman	34	RRMS	48	Natalizumab	1.0	0.16	Negative
Man	32	SPMS	156	Natalizumab	5.5	0.16	Negative
Man	59	RRMS	120	Natalizumab	1.5	0.16	Negative
Woman	58	RRMS	168	Natalizumab	1.0	0.16	Negative
Man	58	RRMS	96	Natalizumab	3.0	0.16	Negative
Woman	34	RRMS	84	Natalizumab	6.5	0.17	Negative
Woman	43	RRMS	228	Teriflunomide	0.0	0.17	Negative
Woman	40	RRMS	156	Natalizumab	6.5	0.17	Negative
Woman	55	RRMS	204	Natalizumab	6.0	0.18	Negative
Man	42	RRMS	36	Natalizumab	1.0	0.18	Negative
Man	41	RRMS	72	Natalizumab	5.0	0.18	Negative
Woman	26	RRMS	72	Natalizumab	1.0	0.19	Negative
Mean	42.74 ± 12.2		137.80 ± 85.12			0.25 ± 0.052	
Median	42 (66–25)		120 (336–24)			0.13 (0.19–0.07)	

RRMS, Relapsing–Remitting Multiple Sclerosis; SPMS, Secondary Progressive Multiple Sclerosis.

**Table 4:** Patients with an intermediate anti-JCV antibody classification.

Sex	Age in Years	Type of MS	Evolution in Months	Current Treatment	EDSS	JCV Index	
Woman	40	RRMS	156	Natalizumab	0.0	0.2	Indeterminate
Woman	32	RRMS	240	Natalizumab	7.0	0.2	Indeterminate
Woman	30	RRMS	204	Natalizumab	1.5	0.21	Indeterminate
Woman	29	RRMS	96	Natalizumab	3.5	0.22	Indeterminate
Man	28	RRMS	84	Natalizumab	0.0	0.22	Indeterminate
Woman	41	RRMS	72	Natalizumab	5.5	0.23	Indeterminate
Woman	47	RRMS	228	Natalizumab	6.5	0.23	Indeterminate
Woman	38	SPMS	24	Natalizumab	6.5	0.24	Indeterminate
Man	37	RRMS	108	Natalizumab	1.5	0.25	Indeterminate
Man	22	RRMS	84	Natalizumab	0.0	0.26	Indeterminate
Woman	37	RRMS	72	Natalizumab	1.5	0.27	Indeterminate
Woman	59	RRMS	228	Fingolimod	6.5	0.3	Indeterminate
Woman	49	RRMS	55	Natalizumab	6.0	0.36	Indeterminate
Man	37	RRMS	72	Glatiramer *	6.0	0.37	Indeterminate
Mean	37.57 ± 9.58		123.07 ± 73.1			0.254 ± 0.052	
Median	37 (59–22)		90 (240–24)			0.23 (0.37–0.2)	

* Glatiramer acetate.

**Table 5:** Patients with a positive anti-JCV antibody classification.

Sex	Age in Years	Type of MS	Evolution in Months	Current Treatment	EDSS	JCV Index	
Woman	38	RRMS	48	Natalizumab	3.0	0.41	Positive
Woman	34	RRMS	156	Alemtuzumab	6.0	0.51	Positive
Woman	49	RRMS	264	Natalizumab	1.5	0.51	Positive
Woman	33	RRMS	36	Natalizumab	1.0	0.58	Positive
Woman	29	RRMS	24	Natalizumab	4.5	0.62	Positive
Woman	34	RRMS	72	Natalizumab	1.5	0.83	Positive
Man	31	RRMS	228	Natalizumab	4.0	0.89	Positive
Woman	41	RRMS	55	Natalizumab	6.5	0.9	Positive
Man	43	RRMS	48	Natalizumab	5.5	0.91	Positive
Woman	46	SPMS	204	Natalizumab	6.5	0.92	Positive
Woman	56	RRMS	84	Natalizumab	3.0	1	Positive
Woman	40	RRMS	120	Natalizumab	6.5	1.03	Positive
Woman	35	RRMS	36	Natalizumab	1.5	1.14	Positive
Woman	42	RRMS	27	Natalizumab	1.5	1.14	Positive
Man	56	RRMS	252	Natalizumab	0.0	1.16	Positive
Man	37	SPMS	144	Natalizumab	7.5	1.27	Positive
Man	28	RRMS	132	Glatiramer *	0.0	1.43	Positive
Woman	32	RRMS	72	Natalizumab	2.0	1.48	Positive
Woman	41	RRMS	156	Cladribine	4.0	1.63	Positive
Woman	60	RRMS	120	Teriflunomide	4.0	1.71	Positive
Woman	34	RRMS	216	Natalizumab	1.5	1.79	Positive
Man	53	SPMS	360	Natalizumab	8.0	1.8	Positive
Man	51	RRMS	384	Natalizumab	6.5	1.86	Positive
Man	60	SPMS	228	Natalizumab	6.5	1.92	Positive
Woman	47	SPMS	360	Natalizumab	6.0	1.92	Positive
Man	42	RRMS	120	Natalizumab	1.5	1.96	Positive
Woman	64	RRMS	228	Cladribine	6.5	1.99	Positive
Woman	56	RRMS	408	Natalizumab	5.5	2	Positive
Woman	33	RRMS	120	Natalizumab	4.5	2.07	Positive
Woman	39	RRMS	36	Natalizumab	6.5	2.08	Positive
Man	46	RRMS	156	Natalizumab	5.0	2.1	Positive
Woman	46	RRMS	120	Natalizumab	7.0	2.1	Positive
Woman	38	RRMS	264	Teriflunomide	5.5	2.12	Positive
Man	44	RRMS	84	Natalizumab	1.5	2.14	Positive
Woman	58	RRMS	192	Immunoglobulin G	9.0	2.15	Positive
Man	40	RRMS	192	Teriflunomide	5.0	2.26	Positive
Woman	47	RRMS	288	Natalizumab	3.0	2.5	Positive
Woman	54	RRMS	108	Natalizumab	6.0	2.5	Positive
Man	46	RRMS	252	Teriflunomide	7.5	2.56	Positive
Man	44	SPMS	228	Natalizumab	7.5	2.61	Positive
Man	48	RRMS	144	Teriflunomide	3.0	2.62	Positive
Woman	52	RRMS	300	Natalizumab	1.5	2.75	Positive
Woman	29	SPMS	204	Natalizumab	8.0	2.9	Positive
Woman	45	RRMS	132	Natalizumab	1.0	2.92	Positive
Woman	27	RRMS	36	Glatiramer *	1.0	2.94	Positive
Man	38	PPMS	96	Fingolimod	6.5	3	Positive
Woman	43	RRMS	96	Natalizumab	6.5	3.1	Positive
Woman	49	RRMS	60	Ocrelizumab	3.0	3.15	Positive
Woman	33	SPMS	144	Alemtuzumab	7.0	3.2	Positive
Man	41	RRMS	72	Fingolimod	1.0	3.2	Positive
Woman	31	RRMS	180	Natalizumab	2.0	3.26	Positive
Man	61	RRMS	132	Natalizumab	3.3	3.26	Positive
Man	30	RRMS	84	Natalizumab	1.0	3.27	Positive
Woman	59	RRMS	276	Fingolimod	6.5	3.27	Positive
Man	37	RRMS	48	Natalizumab	6.5	3.32	Positive
Man	52	RRMS	288	Teriflunomide	6.0	3.34	Positive
Man	22	RRMS	50	Teriflunomide	1.0	3.34	Positive
Man	68	SPMS	300	Natalizumab	8.0	3.35	Positive
Man	59	SPMS	120	Alemtuzumab	6.0	3.36	Positive
Woman	30	RRMS	60	Teriflunomide	2.0	3.38	Positive
Man	52	SPMS	144	Natalizumab	7.0	3.4	Positive
Woman	33	RRMS	36	Fingolimod	4.0	3.44	Positive
Man	50	SPMS	156	Teriflunomide	6.0	3.45	Positive
Woman	52	RRMS	84	Teriflunomide	2.5	3.45	Positive
Woman	40	RRMS	72	Fingolimod	6.5	3.46	Positive
Woman	32	RRMS	132	Alemtuzumab	6.0	3.46	Positive
Man	40	RRMS	120	Fingolimod	5.0	3.49	Positive
Man	55	SPMS	36	Fingolimod	7.0	3.53	Positive
Woman	34	SPMS	109	Fingolimod	4.0	3.57	Positive
Woman	34	RRMS	96	Natalizumab	1.0	3.6	Positive
Woman	42	SPMS	204	Natalizumab	7.5	3.62	Positive
Man	36	RRMS	120	Teriflunomide	1.0	3.66	Positive
Woman	41	SPMS	228	Fingolimod	7.0	3.73	Positive
Woman	46	SPMS	192	Teriflunomide	6.0	3.73	Positive
Man	46	RRMS	252	Natalizumab	1.5	3.78	Positive
Man	31	PPMS	180	Fingolimod	6.0	3.93	Positive
Mean	43.12 ± 10.1		152.33 ± 93.37			2.38 ± 1.02	
Median	22 (68–22)		132 (408–24)			2.53 (3.93–0.41)	

* Glatiramer acetate; RRMS, Relapsing–Remitting Multiple Sclerosis; SPMS, Secondary Progressive Multiple Sclerosis; PPMS, Primary Progressive Multiple Sclerosis.

**Table 6:** Association and correlation of IgG positivity and JCV index with clinical conditions.

Clinical Condition	Type of Analysis	R (Pearson)	*p*-Value
Cognitive impairment	Association	--	0.047
History of depression	Association	--	0.040
Absence of other neurological disease	Association	--	0.041
History of depression	Correlation	−0.202	0.020
Duration of fingolimod use	Correlation	0.480	0.030

## Data Availability

The original contributions presented in this study are included in the article; further inquiries can be directed to the corresponding author.
